# *PBRM1* mutation and preliminary response to immune checkpoint blockade treatment in non-small cell lung cancer

**DOI:** 10.1038/s41698-020-0112-3

**Published:** 2020-03-16

**Authors:** Huaqiang Zhou, Jiaqing Liu, Yaxiong Zhang, Yan Huang, Jiayi Shen, Yunpeng Yang, Wenfeng Fang, Li Zhang

**Affiliations:** 1 Department of Medical Oncology, Sun Yat-sen University Cancer Center, Guangzhou, China; 2 State Key Laboratory of Oncology in South China, Guangzhou, China; 3 Collaborative Innovation Center for Cancer Medicine, Guangzhou, China; 4 Zhongshan School of Medicine, Sun Yat-sen University, Guangzhou, China

**Keywords:** Predictive markers, Non-small-cell lung cancer, Cancer genomics

## Abstract

Polybromo-1 (*PBRM1*) gene is a promising biomarker for immunotherapy in clear cell renal cell carcinoma. But to our knowledge, the frequency and clinical relevance of *PBRM1* mutation in lung cancer remain unknown. We conducted a retrospective study to evaluate the prevalence of *PBRM1* mutation and its correlation with preliminary response to immunotherapy in non-small cell lung cancer (NSCLC). Our results indicated that *PBRM1* mutation was more likely to be a negative predictive biomarker for immunotherapy in NSCLC.

## Introduction

Immune checkpoint blockade (ICB) therapy has been a pivotal treatment for lung cancer^1,2^. However, the response rate of cancer immunotherapy among lung cancer patients is still limited. Although several predictive biomarkers have been identified, such as PD-L1 expression, tumor mutation burden (TMB), and microsatellite instability, additional biomarkers should be found out to cover more patients who may benefit most from ICB therapy^3–5^.

Polybromo-1 (*PBRM1*), located on chromosome 3p21, is a tumor suppressor gene in many cancer types^6^. The existing knowledge regarding its function includes the control of cell cycle, promotion of genomic stability, apoptosis, centromeric cohesion, and so on^7^. Somatic mutations of *PBRM1* are especially prevalent in clear cell renal cell carcinoma (ccRCC)^8^. Previous studies have found that *PBRM1* mutation was a promising biomarker for immunotherapy in ccRCC^9,10^. Strong enrichment of immunostimulatory genes (including genes involved in hypoxia response and JAK–STAT signaling) may be the potential mechanism to enhance the response to ICB therapy in *PBRM1*-deficient ccRCC^9^. Compared with patients without the loss of *PBRM1*, patients with *PBRM1* loss had longer overall survival (OS) and progression-free survival (PFS) (log-rank test *P* = 0.0074 and 0.029, respectively)^9^. To our knowledge, the frequency and clinical relevance of *PBRM1* mutation in lung cancer remain unknown. Therefore, we conducted a retrospective study to evaluate the prevalence of *PBRM1* mutation and its correlation with preliminary response to ICB therapy in non-small cell lung cancer (NSCLC).

## Results

### Patient characteristics

In the 2767 patients included in our study (Supplementary Fig. 1), *PBRM1* mutation was identified in 84 NSCLC patients (3.04%, Supplementary Table 1). Fifty-one patients were found to have *PBRM1* loss-of-function (LOF) mutation, accounting for 60.17% of the mutated patients (Supplementary Fig. 2). Among the 84 mutated patients, 56 (66.67%) had lung adenocarcinoma, and 23 (27.38%) had lung squamous cell carcinoma. The ratio of gender was balanced in this cohort (Male, 43, 51.19%; Female, 41, 48.81%). No significant difference in smoking status was observed between patients with *PBRM1* mutation type (MT) and *PBRM1* wild type (WT).

### *PBRM1* mutation predicts worse response to immunotherapy in NSCLC

**Fig. 1:**
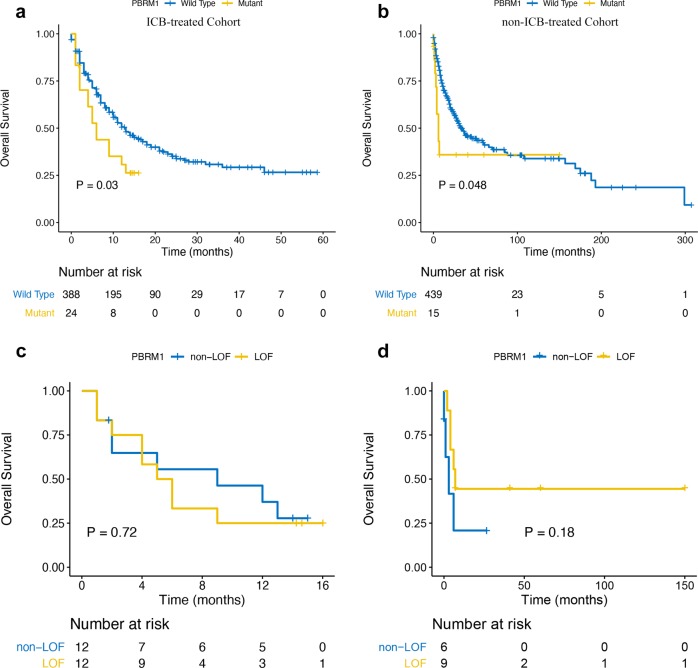
Kaplan–Meier curve comparing overall survival of patients whose tumors did or did not harbor *PBRM1* mutations. **a** The OS of the *PBRM1*-mutant patients was worse than that of those without the mutation in the cohort of ICB-treated patients. **b** There is marginally significant difference in OS between the *PBRM1* mutation subgroup and the *PBRM1* wild type subgroup in the cohort of non-ICB-treated patients. No survival difference between *PBRM1* mutation types (LOF mutation and non-LOF mutation) were observed in the cohort of ICB-treated (**c**) and non-ICB-treated (**d**) patients. OS overall survival, ICB immune checkpoint blockade, LOF loss of function.

**Fig. 2:**
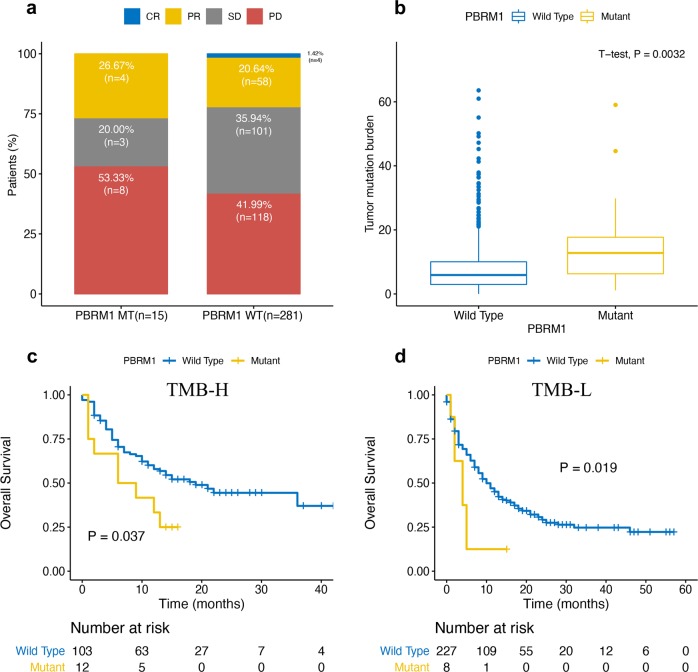
*PBRM1* mutation and response to ICB therapy. **a** *PBRM1* mutation and response to ICB therapy by 296 patients. **b** The boxplot showed that TMB was significantly higher in *PBRM1*-mutated patients. The boundary of the box closest to zero indicates the 25th percentile, a line within the box marks the median, and the boundary of the box farthest from zero indicates the 75th percentile. The whiskers left and right of the box indicate the 90th and 10th percentiles. Points above and below the whiskers indicate outliers outside the 10th and 90th percentiles. Kaplan–Meier curve comparing overall survival of patients whose tumors did or did not harbor *PBRM1* mutations in the TMB-High (**c**) and TMB-Low (**d**) subgroups. ICB immune checkpoint blockade, TMB, tumor mutation burden.

A combined cohort of 441 ICB-treated patients (385 from Memorial Sloan Kettering Cancer Center (MSKCC), 56 from Dana Farber Cancer Institute (DFCI)) were further analyzed to access the association between *PBRM1* mutation and response to ICB therapy. As shown in Table 1, there was no significant difference in the distribution of gender, age, smoking status, and pathology between the two groups (*P* > 0.05). Most of the patients received anti-PD1/PD-L1 monotherapy, and the mean lines of treatment was about 2.3. In the cohort of ICB-treated patients (*N*_os_ = 412, *PBRM1* MT = 24), the OS of the *PBRM1*-mutant patients was worse than that of those without the mutation (*P* = 0.03; Fig. 1a). The median OS of the 24 *PBRM1*-mutant patients was 6 months from the start of ICB therapy, while the median OS of the ICB-treated patients with *PBRM1* WT was 13 months. To further investigate the role of *PBRM1* mutation, we performed the multivariate Cox regression analysis including covariates (mono vs. combo therapy, lines of treatment, smoking, sex, age) using a 211 patients’ subgroup with available data. We found that the *PBRM1* mutation was still negatively associated with poor OS (hazard ratio 2.16, 95% confidence interval 1.03–4.51, *P* = 0.041) after adjusting these covariates. A subgroup of 296 patients from ICB-treated cohort (*N* = 441) was with available data for the evaluation of response to ICB therapy (PFS, objective response rate (ORR), disease control rate (DCR) and durable clinical benefit (DCB)). Among them, 15 patients were detected with *PBRM1* mutation. The median PFS was 2.1 months. The ORR was 26.67%, the DCR was 46.67%, and the DCB was 13.33% (Fig. 2a). In the cohort of non-ICB-treated patients (*N*_os_ = 454, *PBRM1* MT = 15), there seems to be marginally significant difference in OS between the *PBRM1* mutation subgroup and the *PBRM1* WT subgroup, with the survival curves overlapped visually (*P* = 0.048; Fig. 1b).

**Table 1:** The baseline characteristics of 441 ICB-treated patients.

Characteristics	PBRM1 wild type (*N* = 415)	PBRM1 mutant (*N* = 26)	*P* value
Sex (%)			0.207
Male	194 (46.7)	16 (61.5)	
Female	221 (53.3)	10 (38.5)	
Age (%)			0.199
<31	1 (0.3)	0 (0)	
31–50	36 (9.9)	1 (4.3)	
50–60	80 (22.1)	2 (8.7)	
61–70	124 (34.3)	7 (30.4)	
>71	121 (33.4)	13 (56.5)	
NA	53	3	
Smoke (%)			0.133
Never	60 (21.4)	0 (0)	
Ever	208 (74.0)	14 (93.3)	
Current	13 (4.6)	1 (6.7)	
NA	134	11	
Pathology (%)			0.697
LUAD	323 (77.8)	21 (80.8)	
LUSC	54 (13.0)	2 (7.7)	
Other	38 (9.2)	3 (11.5)	
Therapy (%)			0.210
Mono	377 (90.8)	26 (100)	
Combo	38 (9.2)	0 (0)	
Lines of treatment (mean (SD))	2.24 (1.15)	2.33 (0.89)	0.775

In addition, we also observed that there was no survival difference regarding MTs (LOF mutation and non-LOF mutation), when considering the OS in the ICB and non-ICB-treated cohort (Fig. 1c, d).

TMB was significantly higher in *PBRM1*-mutated patients compared with that in *PBRM1* WT patients (median 12.79 and 5.9, respectively, *P* < 0.05, Fig. 2b). In the TMB-high and TMB-low subgroup analysis, we still observed the worse OS in the *PBRM1*-mutant patients treated with ICB therapy (*P* < 0.05, Fig. 2c, d).

## Discussion

In this retrospective study, we combined data from three institutions to investigate the clinical efficacy of ICB therapy in NSCLC patients with or without *PBRM1* mutation. Unlike ccRCC, NSCLC seemed to follow a different *PBRM1* mutation pattern. The prevalence of *PBRM1* mutation (NSCLC: 84/2767, 3.04%; ccRCC: 162/402, 40.30% in The Cancer Genome Atlas (TCGA)) and the proportion of truncating mutation (NSCLC: 51/84, 60.17%; ccRCC: 144/162, 93.51% in TCGA) were relatively low in NSCLC (Supplementary Fig. 2). Our findings suggested that *PBRM1*-mutant NSCLC patients might get less survival benefit from ICB therapy, unlike previously reported data in ccRCC. Interestingly, *PBRM1*-mutant patients tended to have higher TMB. But no matter in TMB-high subgroup or in TMB-low subgroup, *PBRM1*-mutant patients who received ICB therapy had worse survival than those without *PBRM1* mutation. Besides, *PBRM1* mutation was not a remarkable prognostic factor in NSCLC patients according to our analysis in non-ICB-treated patients. Taken together, our results indicated that *PBRM1* was more likely to be a negative predictive biomarker for ICB therapy in NSCLC.

To our knowledge, our study was the first study to estimate the role of *PBRM1* mutation in both ICB and non-ICB-treated NSCLC cohorts. However, due to data restrictions, not all patients have the full record of clinical data. There was discrepancy in the patients when performing different analysis. We were also not able to include PD-L1 level, microsatellite instability and other factors that might influence the response to ICB therapy in our analysis. In addition, the number of *PBRM1*-mutated patients was limited, this low frequency may limit the utility of *PBRM1* mutation as a predictive biomarker, and we still have to interpret the results with caution. Moreover, *PBRM1* mutation did not help predict benefit from the first-line ICB treatment for ccRCC. Most patients received ICB therapy as second or later-line therapy in our cohort. It is still unknown whether *PBRM1* mutation can be a predictive biomarker for the first-line ICB therapy. Therefore, further prospective research is warranted to confirm the negative predictive role of *PBRM1* in NSCLC ICB therapy.

## Methods

### Patients

We analyzed the combined NSCLC cohort of 2767 patients, from three sources: (1) TCGA (*N* = 1144), (2) MSKCC (*N* = 1567), and (3) DFCI (*N* = 56)^5,11–14^.

### *PBRM1* mutation

We first estimated the prevalence of *PBRM1* mutation in the whole NSCLC cohort. *PBRM1* mutation was defined as any SNV or indel, including putative truncating mutations (nonsense mutations, frameshift, insertions and deletions, and splice-site mutations) and other alterations presumed not to be truncating (In-frame insertions and deletions, missense mutations etc.). Notably, homozygous deletion was also calculated in the *PBRM1* mutation. Moreover, we classified *PBRM1* mutations into two type: LOF (any truncating mutation and homozygous deletion) and non-LOF.

### *PBRM1* mutation and response to immunotherapy

A subset of ICB-treated patients (*N* = 441, 385 from MSKCC, 56 from DFCI) with annotated clinical records were further analyzed for the association between *PBRM1* mutation and response to ICB therapy. The OS, PFS (calculated from the date of first ICB infusion) and response to ICB therapy (ORR, DCR, and DCB) were evaluated among these 441 ICB-treated patients. We also calculated the OS (calculated from the date of first chemotherapy infusion) of 454 non-ICB-treated patients from MSKCC cohort. The results of subgroup analysis were also displayed according to the status of *PBRM1* LOF mutations. In addition, we investigated the relationship between TMB and *PBRM1* mutation status (804 available patients, 454 non-ICB; 350 ICB). In order to further clarify the role of *PBRM1* mutation, we classified the ICB-treated patients into two groups (TMB-High and TMB-low, cut-off data: TMB = 10 mut/Mb), and compared their OS. We also showed the PBRM1 mutation landscape of 402 patients with ccRCC from the TCGA database. Institutional review board approval and informed consent were waived because all data were de-identified and publicly available.

### Statistical analysis

Patients’ characteristics at baseline and response to therapy were compared by T test or Mann–Whitney U test (continuous variables) and Pearson chi‐squared test (categorical variables). Kaplan–Meier curve was used to describe the OS and PFS, and the differences between groups were tested by log‐rank method. All statistical analyses were performed using R version 3.5.3 software (Institute for Statistics and Mathematics, Vienna, Austria; www.r-project.org). Statistical significance was set at two-sided *P* < 0.05.

## Supplementary information

reporting-summary

Supplementary Information

## Data Availability

The data that support the findings of this study are available from the website [cBioPortal for Cancer Genomics] (https://www.cbioportal.org/), and are also available from the corresponding author on reasonable request. *TCGA*: Pan-Lung Cancer^11^ [https://www.cbioportal.org/study/summary?id=nsclc_tcga_broad_2016] *MSKCC*: MSK-IMPACT Clinical Sequencing Cohort^12^ [https://www.cbioportal.org/study/summary?id=msk_impact_2017] *DFCI*: MSS Mixed Solid Tumors^13^ [https://www.cbioportal.org/study/summary?id=mixed_allen_2018].

## Code availability

The code that support the findings of this study are available from the corresponding author on reasonable request.
