# Supplementary material for: PBRM1 mutation and preliminary response to immune checkpoint blockade treatment in non-small cell lung cancer
Source: NPJ Precis Oncol. 2020 Mar 16;4:6. doi: 10.1038/s41698-020-0112-3 (PMC7075870; doi:10.1038/s41698-020-0112-3)
Supplement: Supplementary file 2 — Supplementary Information [file 41698_2020_112_MOESM2_ESM.pdf]

PBRM1 mutation and preliminary response to immune checkpoint blockade treatment in non-small cell lung cancer

Supplementary Information

Supplementary Figure 1. The workflow of this study.

Supplementary Figure 2. The *PBRM1* mutation landscape of Non-small cell lung cancer (NSCLC, 2767 patients in our study) and clear cell renal carcinoma (ccRCC, 402 patients from TCGA).

Supplementary Table 1. The *PBRM1* variants within our study cohort (81 mutation and 9 homozygous deletion among 84 *PBRM1* mutated patients).

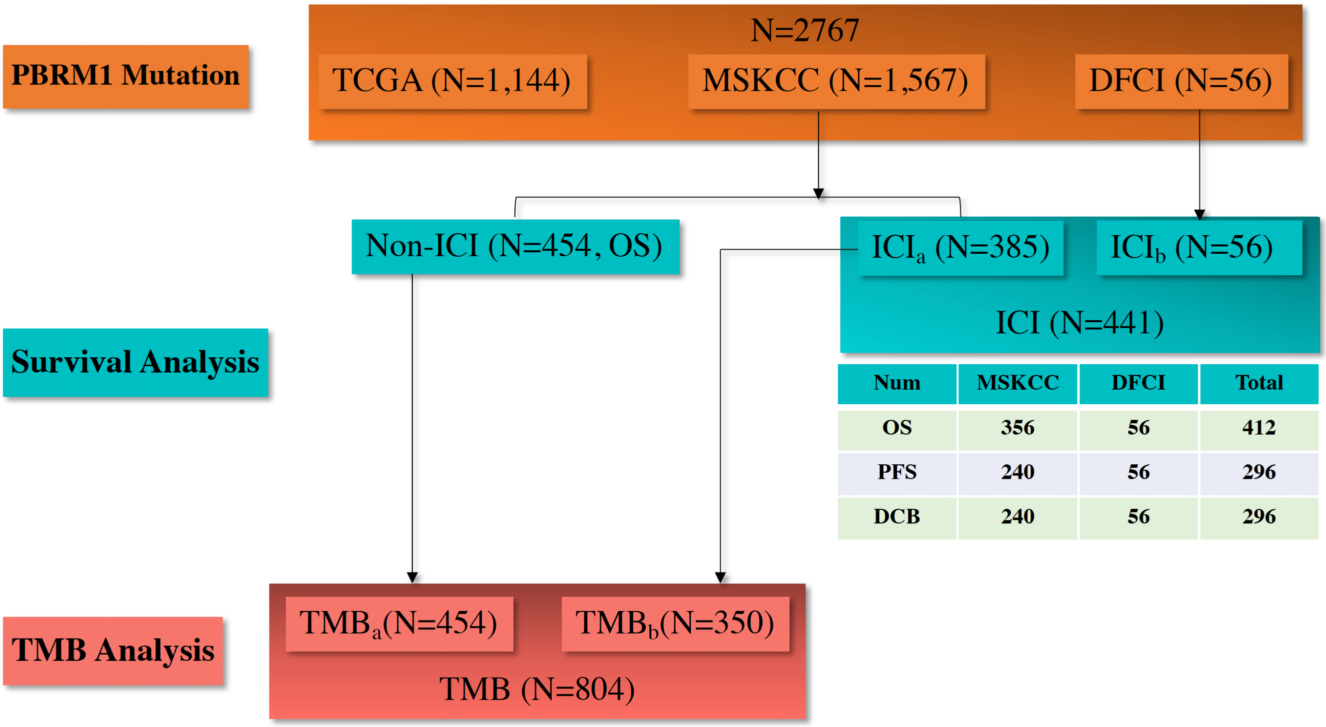

Num	MSKCC	DFCI	Total
OS	356	56	412
PFS	240	56	296
DCB	240	56	296

Supplementary Figure 1. The workflow of this study.

1) *PBRM1* mutation: the prevalence of *PBRM1* mutation in the combined NSCLC cohort of 2767 patients, from 3 sources: (1) The Cancer Genome Atlas (TCGA) (N=1144), (2) Memorial Sloan Kettering Cancer Center (MSKCC) (N=1567), and (3) Dana Farber Cancer Institute (DFCI) (N=56). 2) Survival analysis: A subset of ICB-treated patients (N=441, 385 from MSKCC, 56 from DFCI) and 454 non-ICB treated patients with annotated clinical records were further analyzed for association between *PBRM1* mutation and response to ICB therapy. 3)TMB analysis: we investigated the relationship between TMB and *PBRM1* mutation status (804 available patients from MSKCC, 454 non-ICB; 350 ICB). ICB, immune checkpoint blockade. TMB, Tumor mutation burden.

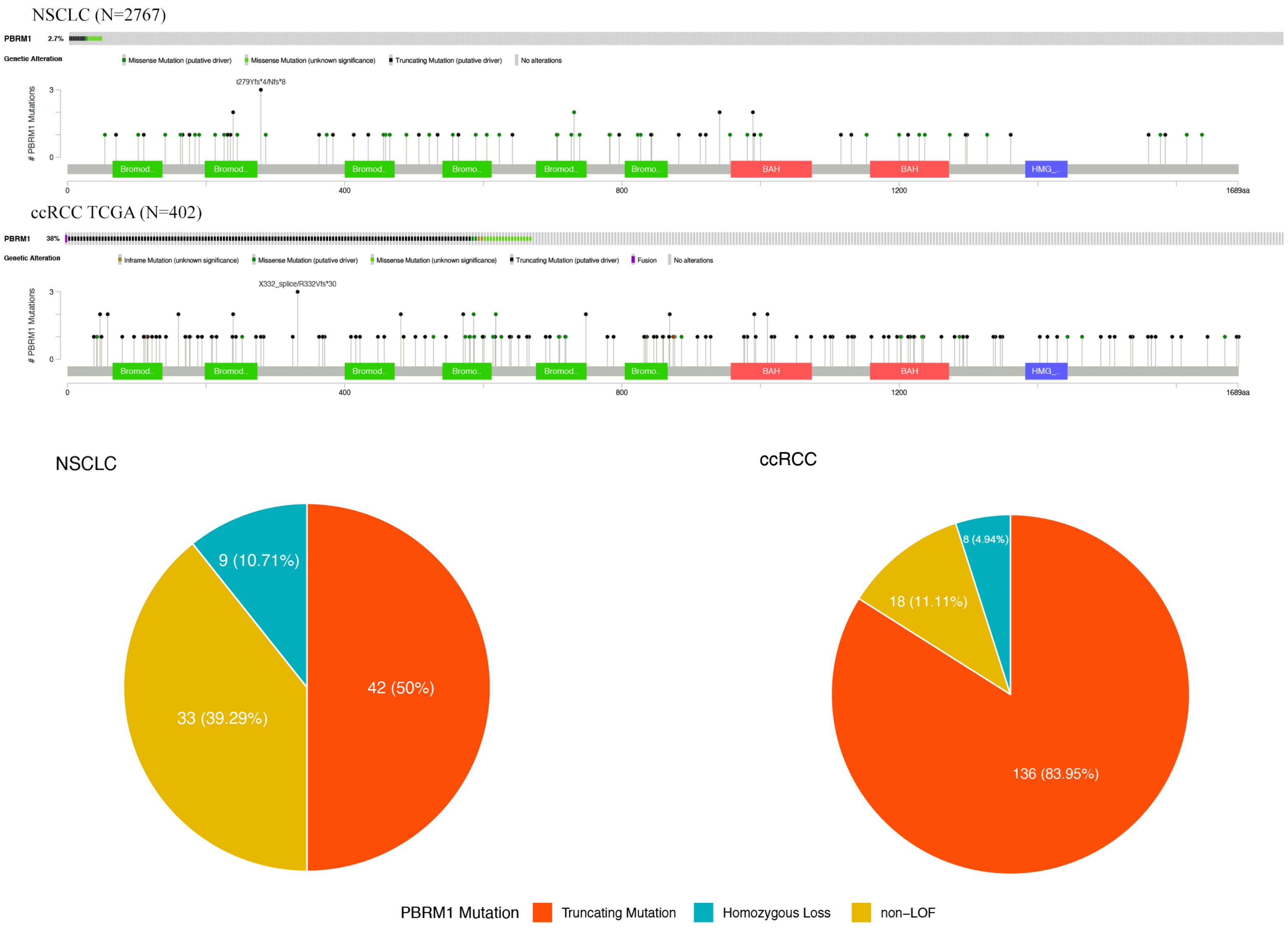

Supplementary Figure 2. The *PBRM1* mutation landscape of Non-small cell lung cancer (NSCLC, 2767 patients in our study) and clear cell renal carcinoma (ccRCC, 402 patients from TCGA). NSCLC seem to follow a different *PBRM1* mutation pattern with ccRCC. The prevalence of PBRM1 mutation (NSCLC: 84/2767, 3.04%; ccRCC: 162/402, 40.30% in TCGA) and the proportion of truncating mutation (NSCLC: 51/84, 60.17%; ccRCC: 144/162, 93.51% in TCGA) were relatively low in NSCLC.

Supplementary Table 1. The PBRM1 variants within our study cohort (81 mutation among 75 PBRM1 mutated patients)

Cohort	Sample ID	Protein Change	Mutation Type	Start Pos	End Pos	Ref	Var	Allele Freq (T)
MSKCC	P-0000168-T01-IM3	X239_splice	Splice_Site	52682459	52682459	C	G	0.06
MSKCC	P-0000491-T01-IM3	X434_splice	Splice_Site	52668617	52668617	C	G	0.3
MSKCC	P-0000626-T01-IM3	R534*	Nonsense_Mutation	52651496	52651496	G	A	0.1
MSKCC	P-0000671-T01-IM3	E796*	Nonsense_Mutation	52643510	52643510	C	A	0.61
MSKCC	P-0000671-T01-IM3	E1327K	Missense_Mutation	52597406	52597406	C	T	0.6
MSKCC	P-0000676-T01-IM3	P556S	Missense_Mutation	52651430	52651430	G	A	0.37
MSKCC	P-0000676-T01-IM3	S605F	Missense_Mutation	52651282	52651282	G	A	0.37
MSKCC	P-0000825-T01-IM3	S413*	Nonsense_Mutation	52668681	52668681	G	C	0.25
MSKCC	P-0001130-T01-IM3	G508Lfs*14	Frame_Shift_Ins	52661309	52661310	-	GTATCAGT	0.23
MSKCC	P-0001189-T01-IM3	E842*	Nonsense_Mutation	52643372	52643372	C	A	0.85
MSKCC	P-0001283-T01-IM3	E1200K	Missense_Mutation	52610650	52610650	C	T	0.07
MSKCC	P-0001365-T02-IM3	L190P	Missense_Mutation	52692291	52692291	A	G	0.06
MSKCC	P-0001550-T01-IM3	D163V	Missense_Mutation	52696189	52696189	T	A	0.15
MSKCC	P-0002016-T01-IM3	S213C	Missense_Mutation	52692222	52692222	G	C	0.12
MSKCC	P-0002085-T01-IM3	N110Ifs*3	Frame_Shift_Del	52702569	52702569	T	-	0.21
MSKCC	P-0002085-T02-IM5	N110Ifs*3	Frame_Shift_Del	52702569	52702569	T	-	0.4
MSKCC	P-0002444-T01-IM3	H843L	Missense_Mutation	52643368	52643368	T	A	0.17
MSKCC	P-0002605-T01-IM3	X642_splice	Splice_Site	52643972	52643972	C	A	0.18
MSKCC	P-0002709-T01-IM3	S383*	Nonsense_Mutation	52668771	52668771	G	C	0.65
MSKCC	P-0003425-T01-IM5	Q1298*	Nonsense_Mutation	52597493	52597493	G	A	0.06
MSKCC	P-0004124-T01-IM5	E1132Rfs*11	Frame_Shift_Ins	52613209	52613210	-	T	0.08
MSKCC	P-0004189-T01-IM5	Q167Kfs*7	Frame_Shift_Del	52696179	52696179	C	-	0.22
MSKCC	P-0004315-T01-IM5	I279Yfs*4	Frame_Shift_Del	52678784	52678784	T	-	0.32
MSKCC	P-0004710-T01-IM5	X363_splice	Splice_Site	52675969	52675969	C	A	0.09
MSKCC	P-0004802-T01-IM5	Y1153C	Missense_Mutation	52613145	52613145	T	C	0.09
MSKCC	P-0005492-T01-IM5	Y54C	Missense_Mutation	52712591	52712591	T	C	0.43
MSKCC	P-0005621-T02-IM5	N882Kfs*19	Frame_Shift_Ins	52637670	52637671	-	T	0.12
MSKCC	P-0005850-T01-IM5	K102E	Missense_Mutation	52702594	52702594	T	C	0.33
MSKCC	P-0005897-T02-IM5	E70Hfs*2	Frame_Shift_Del	52712537	52712544	AAGAGCTC	-	0.41

MSKCC	P-0005975-T01-IM5	I279Nfs*8	Frame_Shift_Ins	52678783	52678784	-	T	0.17
MSKCC	P-0007026-T01-IM5	E374Q	Missense_Mutation	52668799	52668799	C	G	0.06
MSKCC	P-0007525-T01-IM5	P1297Rfs*15	Frame_Shift_Del	52597494	52597497	AGGA	-	0.05
MSKCC	P-0008046-T01-IM5	W141L	Missense_Mutation	52696255	52696255	C	A	0.07
MSKCC	P-0008848-T01-IM5	E1615Q	Missense_Mutation	52584491	52584491	C	G	0.35
MSKCC	P-0008863-T01-IM5	T232Pfs*21	Frame_Shift_Del	52685779	52685779	C	-	0.16
MSKCC	P-0008874-T01-IM5	M731L	Missense_Mutation	52643705	52643705	T	A	0.07
MSKCC	P-0008874-T01-IM5	D727E	Missense_Mutation	52643715	52643715	G	C	0.06
MSKCC	P-0009196-T01-IM5	H783Y	Missense_Mutation	52643549	52643549	G	A	0.08
MSKCC	P-0009295-T01-IM5	E913*	Nonsense_Mutation	52637579	52637579	C	A	0.61
MSKCC	P-0009638-T01-IM5	M706I	Missense_Mutation	52643778	52643778	C	A	0.07
MSKCC	P-0010181-T01-IM5	E286K	Missense_Mutation	52678763	52678763	C	T	0.12
MSKCC	P-0010337-T01-IM5	K827N	Missense_Mutation	52643415	52643415	C	G	0.15
MSKCC	P-0010519-T01-IM5	R921*	Nonsense_Mutation	52637555	52637555	G	A	0.21
MSKCC	P-0010561-T01-IM5	E1214Rfs*6	Frame_Shift_Ins	52610608	52610609	-	T	0.2
MSKCC	P-0010561-T01-IM5	P1584Rfs*31	Frame_Shift_Del	52584582	52584583	AG	-	0.22
MSKCC	P-0011242-T01-IM5	X239_splice	Splice_Site	52682459	52682459	C	T	0.13
MSKCC	P-0011260-T01-IM5	Q235Rfs*18	Frame_Shift_Del	52685769	52685769	G	-	0.42
MSKCC	P-0012001-T01-IM5	D589N	Missense_Mutation	52651331	52651331	C	T	0.11
TCGA	LUAD-CHTN-MAD06-00490-Tumor	G1273A	Missense_Mutation	52598123	52598123	C	G	0.08
TCGA	LUAD-E00443-Tumor	P465S	Missense_Mutation	52662960	52662960	G	A	0.06
TCGA	LUAD-RT-S01702-Tumor	I279Yfs*4	Frame_Shift_Del	52678784	52678784	T	-	0.44
TCGA	LUAD-RT-S01813-Tumor	A459G	Missense_Mutation	52662977	52662977	G	C	0.14
TCGA	LUAD-S01381-Tumor	I782L	Missense_Mutation	52643552	52643552	T	G	0.22
TCGA	LUAD-S01478-Tumor	S1116*	Nonsense_Mutation	52620481	52620481	G	C	0.47
TCGA	LUAD-TJW61-Tumor	R489S	Missense_Mutation	52661363	52661363	C	G	0.08
TCGA	TCGA-05-4382-01	N739S	Missense_Mutation	52643680	52643680	T	C	0.27
TCGA	TCGA-18-3417-01	X989_splice	Splice_Site	52621527	52621527	C	T	0.53
TCGA	TCGA-18-3417-01	E226Q	Missense_Mutation	52685796	52685796	C	G	0.48
TCGA	TCGA-21-1079-01	S956R	Missense_Mutation	52623183	52623183	G	T	0.33
TCGA	TCGA-38-4629-01	R522L	Missense_Mutation	52651531	52651531	C	A	0.33

TCGA	TCGA-39-5024-01	I245F	Missense_Mutation	52682440	52682440	T	A	0.19
TCGA	TCGA-39-5031-01	E184D	Missense_Mutation	52692308	52692308	C	G	0.11
TCGA	TCGA-44-6776-01	M731K	Missense_Mutation	52643704	52643704	A	T	0.06
TCGA	TCGA-44-7659-01	X1560_splice	Splice_Site	52584762	52584762	C	A	0.45
TCGA	TCGA-55-7907-01	S941*	Nonsense_Mutation	52623229	52623229	G	C	0.42
TCGA	TCGA-56-8622-01	E707*	Nonsense_Mutation	52643777	52643777	C	A	0.27
TCGA	TCGA-60-2698-01	D823H	Missense_Mutation	52643429	52643429	C	G	0.33
TCGA	TCGA-60-2703-01	I1229V	Missense_Mutation	52610563	52610563	T	C	0.3
TCGA	TCGA-63-5131-01	D1637N	Missense_Mutation	52582240	52582240	C	T	0.04
TCGA	TCGA-66-2754-01	X989_splice	Splice_Site	52623085	52623085	C	G	0.41
TCGA	TCGA-66-2782-01	F456S	Missense_Mutation	52662986	52662986	A	G	0.29
TCGA	TCGA-67-3770-01	G1577C	Missense_Mutation	52584605	52584605	C	A	0.22
TCGA	TCGA-85-6561-01	R1000G	Missense_Mutation	52621494	52621494	G	C	0.07
TCGA	TCGA-85-8479-01	W992Mfs*12	Frame_Shift_Ins	52621518	52621519	-	T	0.43
TCGA	TCGA-86-6851-01	K623R	Missense_Mutation	52649423	52649423	T	C	0.26
TCGA	TCGA-86-8672-01	S941*	Nonsense_Mutation	52623229	52623229	G	C	0.42
TCGA	TCGA-94-8491-01	P1361Hfs*23	Frame_Shift_Del	52597303	52597303	G	-	0.3
TCGA	TCGA-NJ-A55R-01	E981Q	Missense_Mutation	52623110	52623110	C	G	0.06
DFCI	M4945	X176_splice	Splice_Site	52696148	52696148	C	A	0.32
DFCI	SU2C_Lung-SU2C-DFCI-LUAD-1017-Tumor-SM-AOL99	E564*	Nonsense_Mutation	52651406	52651406	C	A	0.52
DFCI	MA7027	S1237L	Missense_Mutation	52598231	52598231	G	A	0.37

(9 patients with PBRM1 homozygous deletion)

Cohort	Sample ID	Copy-number Alterations
MSKCC	P-0001165-T01-IM3	HOMDEL
MSKCC	P-0003460-T01-IM5	HOMDEL
MSKCC	P-0008406-T01-IM5	HOMDEL
TCGA	LUAD-S01473-Tumor	HOMDEL
TCGA	TCGA-33-6737-01	HOMDEL
TCGA	TCGA-39-5034-01	HOMDEL
TCGA	TCGA-60-2695-01	HOMDEL
TCGA	TCGA-85-A510-01	HOMDEL
TCGA	TCGA-52-7809-01	HOMDEL
